# Comparative predictive value of the cholesterol-high-density lipoprotein-glucose index versus the triglyceride-glucose index for gestational dysglycemia: a two-cohort study

**DOI:** 10.3389/fendo.2026.1801546

**Published:** 2026-04-27

**Authors:** Mingliang Liu, Shihang Chen, Shi Wu, Hang Guo, Liming Chen

**Affiliations:** 1 School of Medicine, Nankai University, Tianjin, China; 2 NHC Key Laboratory of Hormones and Development, Tianjin Key Laboratory of Metabolic Diseases, Chu Hsien-I Memorial Hospital & Tianjin Institute of Endocrinology, Tianjin Medical University, Tianjin, China

**Keywords:** CHG, gestational diabetes mellitus, gestational dysglycemia, NHANES, risk stratification, TyG

## Abstract

**Background:**

Early risk stratification for gestational dysglycemia is important for improving maternal and neonatal outcomes. Derived from fasting triglycerides and glucose, the triglyceride–glucose (TyG) index is widely used to approximate insulin resistance, whereas the cholesterol-high-density lipoprotein-glucose (CHG) index incorporates broader lipid metabolism. We compared the associations and discriminative performance of TyG and CHG in a national survey discovery cohort and an independent clinical validation cohort.

**Methods:**

We analyzed a survey-weighted discovery cohort from NHANES 2007–2018, in which the primary outcome was self-reported GDM history. We further evaluated an independent validation cohort with clinically diagnosed GDM (n = 217). Associations and predictive performance were assessed using multivariable logistic regression, receiver operating characteristic (ROC) analysis, calibration analysis, and decision curve analysis (DCA). Additional analyses included adjustment for continuous fasting blood glucose in NHANES, supportive analyses restricted to currently pregnant NHANES participants from 2007–2012 using proxy-defined gestational fasting dysglycemia (fasting blood glucose ≥5.1 mmol/L), and gestational-week-adjusted sensitivity analyses in the validation cohort.

**Results:**

In the NHANES discovery cohort, CHG showed a stronger association with self-reported GDM history than TyG in the primary adjusted models and yielded a numerically higher AUC than TyG. After additional adjustment for continuous fasting blood glucose, the association for TyG was attenuated, whereas CHG remained significantly associated. In the clinical validation cohort, CHG also showed numerically higher discriminative performance than TyG, and the overall findings remained directionally consistent after gestational-week adjustment. Supportive analyses in currently pregnant NHANES participants showed directionally similar but statistically imprecise estimates because of the limited sample size.

**Conclusion:**

Both TyG and CHG are simple, low-cost indices associated with gestational dysglycemia/GDM. Across the discovery and validation cohorts, CHG generally showed stronger associations and numerically better discrimination than TyG; however, its overall discriminative performance remained modest and should be interpreted as that of a potential risk marker rather than a standalone clinical screening tool. Further prospective studies are needed to validate these findings.

## 1 Introduction

Gestational diabetes mellitus (GDM) is among the most common metabolic complications of pregnancy, with a steadily rising global prevalence and substantial public health impact (1–3). GDM increases the risk of adverse maternal outcomes, including preeclampsia, labor complications, and caesarean delivery, and is associated with lasting cardiometabolic sequelae in the offspring, including greater susceptibility to overweight/obesity, type 2 diabetes, and cardiovascular disease (4–6). Accordingly, early risk assessment and timely intervention are important for improving maternal and infant outcomes.

In clinical practice, early identification of women at elevated risk remains challenging because standard diagnostic testing (e.g., the oral glucose tolerance test [OGTT]) is most often scheduled between 24 and 28 weeks of gestation, whereas metabolic dysregulation often develops gradually from early gestation. This creates a window in early pregnancy during which risk is evolving but definitive diagnosis is not routinely established. As a result, there is an unmet need for simple, cost-effective, and readily available markers that can be applied early using routinely collected clinical information and laboratory biomarkers to support risk stratification and targeted follow-up (7–9). Importantly, such tools are intended to complement—rather than replace—standard diagnostic testing: they may help prioritize individuals for earlier lifestyle counseling, closer monitoring, or timely confirmatory testing, thereby improving the efficiency of preventive care pathways.

Because insulin resistance is central to the pathophysiology of GDM, the triglyceride–glucose (TyG) index, derived from fasting triglycerides and glucose, has been widely studied as a practical indicator of insulin resistance for GDM risk assessment (10–13). However, its reported performance has been inconsistent across studies (10, 14–18). Prior evidence suggests that the utility of TyG may vary with baseline metabolic status, particularly adiposity-related profiles, and may be influenced by coexisting metabolic disturbances, which could limit its generalizability across diverse populations (10, 15–18). Moreover, an index constructed primarily from fasting glycemia and triglycerides may not fully capture the broader metabolic heterogeneity of pregnancy, during which multiple lipid and lipoprotein pathways undergo dynamic physiological adaptations.

The CHG index, which integrates fasting glucose with cholesterol-related metrics (total cholesterol [TC] and high-density lipoprotein cholesterol [HDL-C]), has been proposed as an alternative marker that incorporates additional lipid-related information alongside glycemia. This rationale is supported by accumulating evidence that lipid-derived markers—such as TG/HDL-C and TC/HDL-C ratios—are associated with GDM risk and metabolic abnormalities (19–25). Pregnancy is characterized by progressive changes in lipid metabolism, and dysregulation in lipid handling may occur in parallel with glucose intolerance and insulin resistance. We therefore hypothesized that incorporating cholesterol-related information may provide complementary metabolic information for early gestational dysglycemia/GDM risk assessment; therefore, a direct comparison with TyG was warranted.

Accordingly, the primary objective of this study was to conduct a head-to-head comparison of CHG and TyG across multiple complementary dimensions, including strength of association, dose–response patterns, discrimination performance, and incremental predictive value. To enhance the reliability and generalizability of our findings, we adopted a dual-cohort design. We first evaluated these indices in the U.S. National Health and Nutrition Examination Survey (NHANES) as a discovery cohort and then externally assessed the findings in an independent single-center retrospective clinical cohort from China. This design was intended to provide complementary evidence across populations with different baseline risk profiles and clinical settings. Ultimately, this study aims to provide evidence to inform and refine early risk stratification strategies for gestational dysglycemia/GDM in routine clinical care.

## 2 Methods

### 2.1 Study design and participants

**Figure 1:**
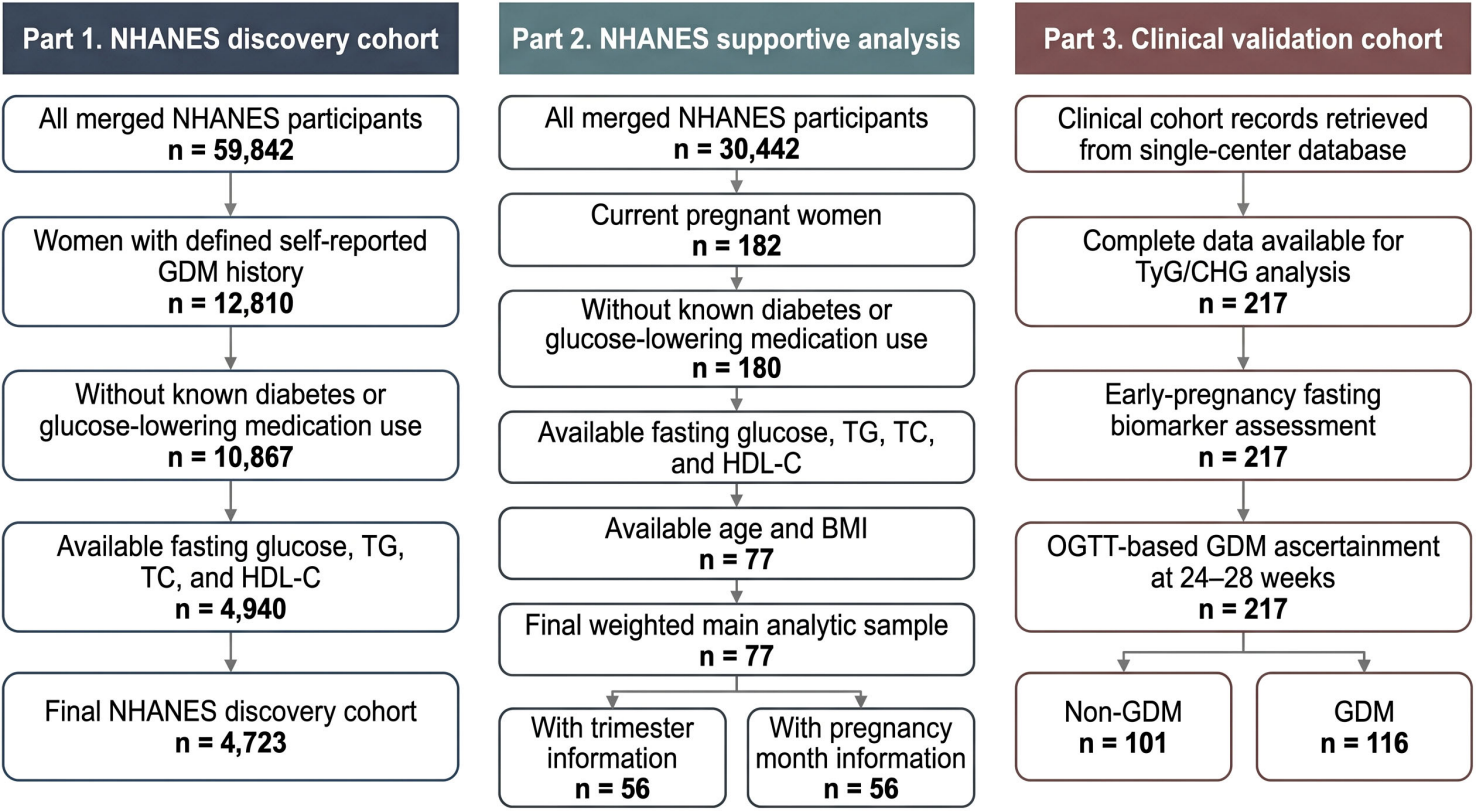
Study design and participant flow.

This study included two independent cohorts: a discovery cohort derived from the U.S. National Health and Nutrition Examination Survey (NHANES) and a retrospective validation cohort from Tianjin Medical University Chu Hsien-I Memorial Hospital (**Figure 1**).

### 2.2 Discovery cohort (NHANES)

NHANES is a continuous, nationally based survey designed to assess the health and nutritional status of the U.S. population through standardized household interviews, physical examinations, and laboratory assessments conducted in mobile examination centers. It collects sociodemographic characteristics, medical history, examination measures, and laboratory biomarkers under standardized protocols. Fasting laboratory measurements are available in designated subsamples.

For the primary discovery analysis, women with available data from NHANES cycles 2007–2018 were included according to the predefined eligibility criteria for the analytic sample. Participants were required to have available measurements for fasting glucose, triglycerides (TG), total cholesterol (TC), and high-density lipoprotein cholesterol (HDL-C), as well as key covariates including age, body mass index (BMI), and blood pressure. Individuals with known pre-existing diabetes, defined as a self-reported physician diagnosis or use of antidiabetic medications, were excluded. The final NHANES discovery cohort included 4,723 participants.

In the primary NHANES analysis, the outcome was self-reported GDM history. Because detailed pregnancy OGTT data are not uniformly available in NHANES, an additional supportive analysis was performed in currently pregnant NHANES participants from 2007–2012. In that supportive analysis, proxy-defined gestational fasting dysglycemia was defined as fasting blood glucose (FBG) ≥5.1 mmol/L (92 mg/dL), consistent with the fasting threshold recommended by the International Association of Diabetes and Pregnancy Study Groups (IADPSG).

All NHANES analyses were conducted using the complex survey design variables, including sampling weights, strata, and primary sampling units, to account for the multistage sampling structure.

### 2.3 Validation cohort

We performed a retrospective cohort study based on the diabetes-specific database within the Big-data Intelligence Platform of Tianjin Medical University Chu Hsien-I Memorial Hospital. The validation cohort was recruited from Tianjin Medical University Chu Hsien-I Memorial Hospital, a tertiary specialized center for metabolic diseases in China. Given the specialized clinical setting, the cohort included a relatively high proportion of metabolically high-risk pregnancies compared with community-based obstetric populations.

Eligible participants were women who registered for antenatal care, underwent fasting blood sampling during routine antenatal evaluation at approximately 12 weeks of gestation for measurement of TyG/CHG component biomarkers, underwent standard screening for GDM using a 75-g oral glucose tolerance test (OGTT) at 24–28 weeks of gestation, and delivered at our hospital between January 2020 and June 2024. Exclusion criteria included pre-existing type 1 or type 2 diabetes, multiple gestations, and incomplete medical records. The final validation cohort included 217 participants, including 116 with OGTT-confirmed GDM and 101 without GDM. Gestational age at blood sampling was recorded and further incorporated as an additional covariate in sensitivity analyses.

### 2.4 Definitions and measurements

In the validation cohort, GDM was diagnosed according to the IADPSG criteria based on a 75-g OGTT performed at 24–28 weeks of gestation. Gestational age at blood sampling refers to the gestational age at which fasting blood samples used to calculate TyG and CHG were obtained. In the primary NHANES cohort, the outcome was self-reported GDM history. In the currently pregnant NHANES supportive analyses, proxy-defined gestational fasting dysglycemia was defined as FBG ≥ 5.1 mmol/L (92 mg/dL).

### 2.5 Index calculation

The TyG index was calculated as ln (TG (mg/dL) × FBG (mg/dL)/2) (14). The CHG index was calculated as ln [TC (mg/dL) × FBG (mg/dL)/(2 × HDL-C (mg/dL))] (13).

### 2.6 Ethical considerations

NHANES data are publicly available. All NHANES participants provided written informed consent, and survey protocols were approved by the National Center for Health Statistics Research Ethics Review Board. The validation cohort study was approved by the Ethics Committee of Tianjin Medical University Chu Hsien-I Memorial Hospital (approval number: ZXYJNYYkMEC2025-47). Written informed consent was waived for the validation cohort because of the retrospective design and the use of de-identified routinely collected clinical records.

### 2.7 Statistical analysis

Baseline characteristics were summarized as mean ± standard deviation (SD) for continuous variables and as counts (percentages) for categorical variables, as appropriate. Between-group comparisons were conducted using Student’s t-test or chi-square test, where appropriate.

TyG and CHG were analyzed as both continuous variables and quartiles. Quartiles were defined within each cohort according to the cohort-specific distribution of each index, with the lowest quartile (Q1) serving as the reference category.

In the NHANES discovery cohort, associations were evaluated using survey-weighted logistic regression models. In the validation cohort, associations were evaluated using multivariable logistic regression models. Progressive adjustment models were specified according to cohort-specific analytic objectives. In the validation cohort, Model 1 was unadjusted, Model 2 adjusted for age and BMI, Model 3 further adjusted for systolic blood pressure (SBP) and diastolic blood pressure (DBP), and Model 4 additionally adjusted for gestational week. Results are presented as odds ratios (ORs) with 95% confidence intervals (CIs).

Dose–response relationships were examined using restricted cubic spline regression. Overall and non-linearity p values were calculated to characterize dose-response patterns.

Discrimination was evaluated using receiver operating characteristic (ROC) curves and the area under the curve (AUC). In the validation cohort, incremental discrimination beyond a clinical baseline model was also assessed. Calibration was assessed using calibration plots and a goodness-of-fit approach. Clinical utility was evaluated using decision curve analysis (DCA) and clinical impact curves (CIC).

Additional sensitivity analyses were performed to address key methodological concerns. In NHANES, we additionally adjusted for continuous FBG to assess the extent to which associations for TyG and CHG were independent of fasting glycemia. We also conducted supportive analyses restricted to currently pregnant NHANES participants, with additional adjustment using trimester or pregnancy month where available. In the validation cohort, gestational-week-adjusted analyses were performed as prespecified sensitivity analyses.

Subgroup analyses were conducted for prespecified variables, and interaction tests were considered exploratory; no formal multiplicity adjustment was applied.

Missingness in the validation cohort was <5% for all variables and was handled using multiple imputation by chained equations. The NHANES analyses were restricted to eligible participants with available data required for index calculation and covariate adjustment. All analyses were conducted in R software (version 4.3.0; R Foundation for Statistical Computing, Vienna, Austria). A two-sided P < 0.05 was considered statistically significant.

## 3 Results

### 3.1 Baseline characteristics of the NHANES discovery cohort

In the NHANES discovery cohort, compared with women without a self-reported history of GDM, those with a self-reported history of GDM were younger and had higher BMI and DBP, whereas SBP was lower (all P < 0.05). Fasting blood glucose was higher in women with a history of GDM (P = 0.009). Among lipid-related markers, HDL-C was lower and CHG index was higher in the history-of-GDM group (both P < 0.001), whereas differences in triglycerides, total cholesterol, and TyG index did not reach statistical significance. With respect to sociodemographic characteristics, marital status differed between groups (P = 0.022), while race/ethnicity, education level, poverty income ratio, smoking status, and alcohol use were not significantly different (all P > 0.05) (**Table 1**).

**Table 1:** Baseline characteristics of participants in the NHANES discovery cohort according to self-reported history of gestational diabetes mellitus.

Variable	Overall (n=4,723)	No history of GDM (n=4,443)	History of GDM (n=280)	P value
Age, years	49.91 (0.32)	50.34 (0.32)	43.31 (0.90)	<0.001
Poverty income ratio	2.91 (0.05)	2.91 (0.05)	2.76 (0.13)	0.274
BMI, kg/m^2^	29.04 (0.17)	28.96 (0.17)	30.40 (0.64)	0.025
SBP, mmHg	119.85 (0.35)	120.13 (0.36)	115.48 (1.00)	<0.001
DBP, mmHg	68.50 (0.30)	68.42 (0.30)	69.88 (0.63)	0.017
Fasting blood glucose, mg/dL	99.55 (0.31)	99.22 (0.26)	104.67 (2.09)	0.009
Triglycerides, mg/dL	110.18 (1.33)	109.63 (1.39)	118.70 (6.11)	0.158
Total cholesterol, mg/dL	199.00 (0.83)	199.26 (0.87)	194.90 (2.55)	0.112
HDL-C, mg/dL	60.20 (0.42)	60.61 (0.43)	53.87 (1.30)	<0.001
TyG index	8.45 (0.01)	8.44 (0.01)	8.54 (0.05)	0.053
CHG index	5.11 (0.01)	5.10 (0.01)	5.24 (0.04)	<0.001
Race/ethnicity				0.074
Mexican American	738 (15.6%)	683 (15.4%)	55 (19.6%)	
Other Hispanic	552 (11.7%)	526 (11.8%)	26 (9.3%)	
Non-Hispanic White	2025 (42.8%)	1920 (43.2%)	105 (37.5%)	
Non-Hispanic Black	927 (19.6%)	875 (19.7%)	52 (18.6%)	
Other Race, including multi-racial	485 (10.3%)	443 (10.0%)	42 (15.0%)	
Education level				0.110
< 9th grade	197 (11.4%)	185 (11.3%)	12 (13.0%)	
9-11th grade	293 (16.9%)	272 (16.6%)	21 (22.8%)	
High school/GED	415 (23.9%)	398 (24.3%)	17 (18.5%)	
Some college/AA	502 (29.0%)	474 (28.9%)	28 (30.4%)	
College graduate or above	326 (18.8%)	312 (19.0%)	14 (15.2%)	
Marital status				0.022
Married	2434 (51.5%)	2265 (50.9%)	169 (60.4%)	
Widowed	557 (11.8%)	550 (12.4%)	7 (2.5%)	
Divorced	644 (13.6%)	608 (13.7%)	36 (12.9%)	
Separated	204 (4.3%)	187 (4.2%)	17 (6.1%)	
Never married	511 (10.8%)	487 (11.0%)	24 (8.6%)	
Living with partner	377 (8.0%)	350 (7.9%)	27 (9.6%)	
Smoking status				0.915
Yes	1767 (37.4%)	1661 (37.4%)	106 (37.9%)	
No	2956 (62.6%)	2782 (62.6%)	174 (62.1%)	
Alcohol use				0.366
Yes	3087 (65.3%)	2902 (65.3%)	185 (66.1%)	
No	1638 (34.7%)	1543 (34.7%)	95 (33.9%)	

Continuous variables are presented as weighted mean (SE). Categorical variables are presented as unweighted counts and column percentages. P values were calculated using survey-weighted tests.

### 3.2 Association and dose–response relationship of TyG and CHG with self-reported GDM history in the NHANES discovery cohort

In the NHANES discovery cohort, survey-weighted logistic regression showed that both TyG and CHG were associated with self-reported GDM history when analyzed as continuous variables (**Table 2**). In the fully adjusted model (Model 3), the OR was 1.61 (95% CI, 1.22–2.13) for TyG and 3.59 (95% CI, 2.22–5.80) for CHG. Similar associations were observed in Model 2, whereas the association for TyG in Model 1 was of borderline significance.

**Table 2:** Weighted associations of TyG and CHG indices with self-reported history of gestational diabetes mellitus in the NHANES discovery cohort.

Categories	Model 1			Model 2			Model 3		
	OR	95%CI	P value	OR	95%CI	P value	OR	95%CI	P value
TyG index									
Continuous	1.33	1.00-1.77	0.050	1.54	1.16-2.03	0.003	1.61	1.22-2.13	0.001
Quartile Q1	Ref	–	–	Ref	–	–	Ref	–	–
Quartile Q2	1.21	0.78-1.87	0.398	1.38	0.88-2.15	0.160	1.62	1.04-2.52	0.034
Quartile Q3	1.29	0.86-1.95	0.222	1.59	1.03-2.48	0.041	1.78	1.16-2.75	0.010
Quartile Q4	1.68	1.05-2.69	0.033	2.2	1.31-3.70	0.004	2.58	1.57-4.24	<0.001
CHG index									
Continuous	2.74	1.72-4.38	<0.001	3.31	2.05-5.34	<0.001	3.59	2.22-5.80	<0.001
Quartile Q1	Ref	–	–	Ref	–	–	Ref	–	–
Quartile Q2	1.12	0.65-1.95	0.679	1.23	0.70-2.16	0.480	1.43	0.82-2.50	0.210
Quartile Q3	1.38	0.86-2.20	0.185	1.63	0.96-2.76	0.074	1.93	1.15-3.24	0.014
Quartile Q4	2.45	1.51-3.98	<0.001	2.91	1.71-4.95	<0.001	3.43	2.04-5.75	<0.001

Data are presented as odds ratios (ORs) with 95% confidence intervals (CIs). TyG and CHG indices were analyzed as continuous variables and as quartiles, with Q1 as the reference group. Model 1, unadjusted. Model 2, adjusted for age and BMI. Model 3, further adjusted for systolic blood pressure and diastolic blood pressure. The NHANES outcome in this analysis was self-reported history of gestational diabetes mellitus.

When analyzed by quartiles, TyG showed progressively higher odds of self-reported GDM history across increasing categories. In the fully adjusted model, compared with Q1, the ORs were 1.62 (95% CI, 1.04–2.52) for Q2, 1.78 (95% CI, 1.16–2.75) for Q3, and 2.58 (95% CI, 1.57–4.24) for Q4. For CHG, the corresponding ORs in Model 3 were 1.43 (95% CI, 0.82–2.50) for Q2, 1.93 (95% CI, 1.15–3.24) for Q3, and 3.43 (95% CI, 2.04–5.75) for Q4.

**Figure 2:**
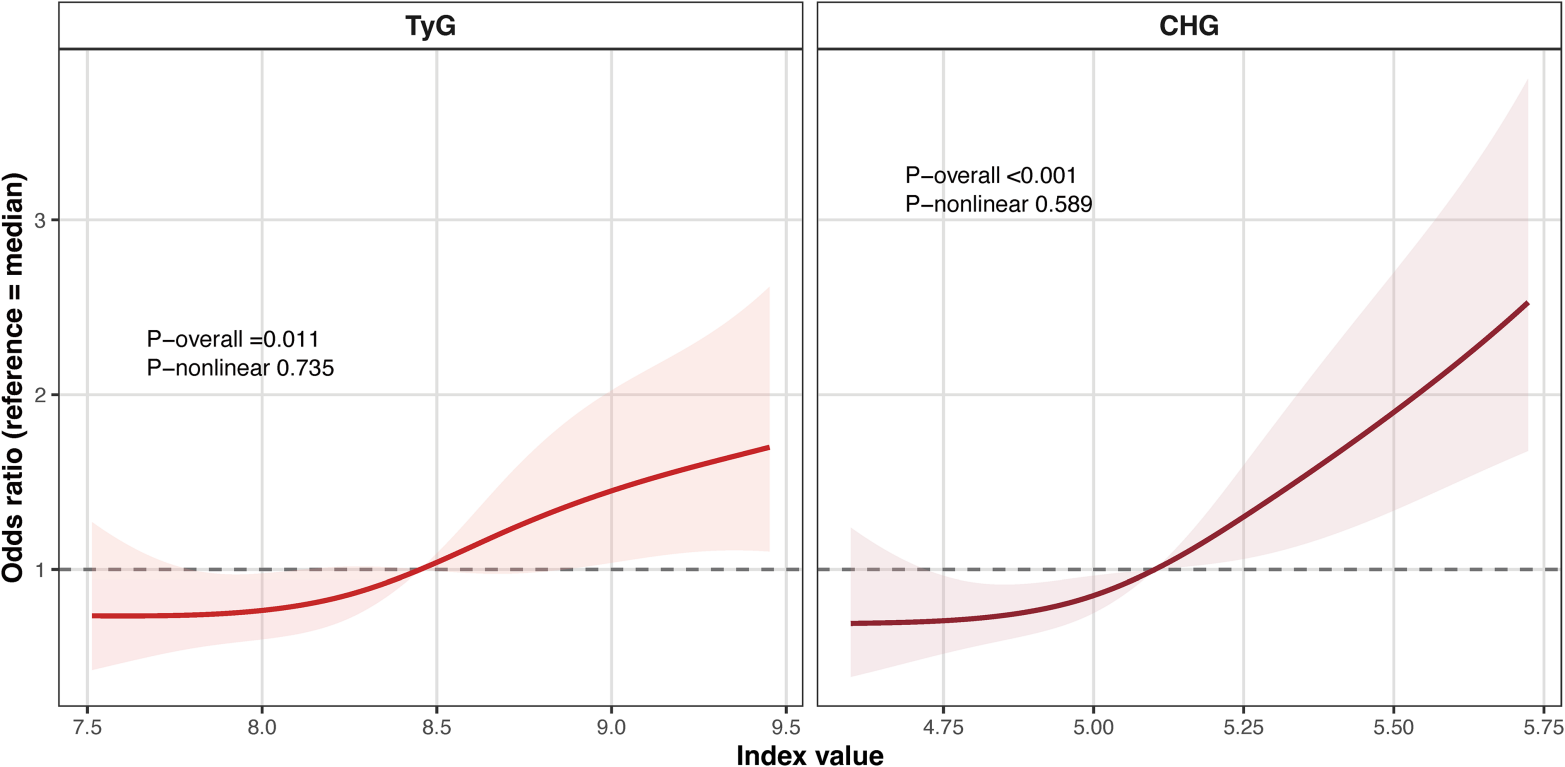
Dose–response relationships of TyG and CHG with self-reported GDM history in the NHANES discovery cohort.Restricted cubic spline curves showing the associations of TyG and CHG with self-reported GDM history. Odds ratios (solid lines) and 95% confidence intervals (shaded areas) were estimated from survey-weighted models. TyG, triglyceride-glucose index; CHG, cholesterol-high-density lipoprotein-glucose index; GDM, gestational diabetes mellitus.

Restricted cubic spline analyses further characterized the dose–response relationships of both indices with self-reported GDM history (**Figure 2**). For TyG, the overall association was statistically significant (P-overall = 0.011), whereas no evidence of nonlinearity was observed (P-nonlinear = 0.735). For CHG, the overall association was also significant (P-overall < 0.001), with no significant evidence of nonlinearity (P-nonlinear = 0.589). Overall, the adjusted odds of self-reported GDM history increased with higher values of both TyG and CHG across the observed ranges.

### 3.3 Discriminative performance of TyG and CHG in the NHANES discovery cohort

**Figure 3:**
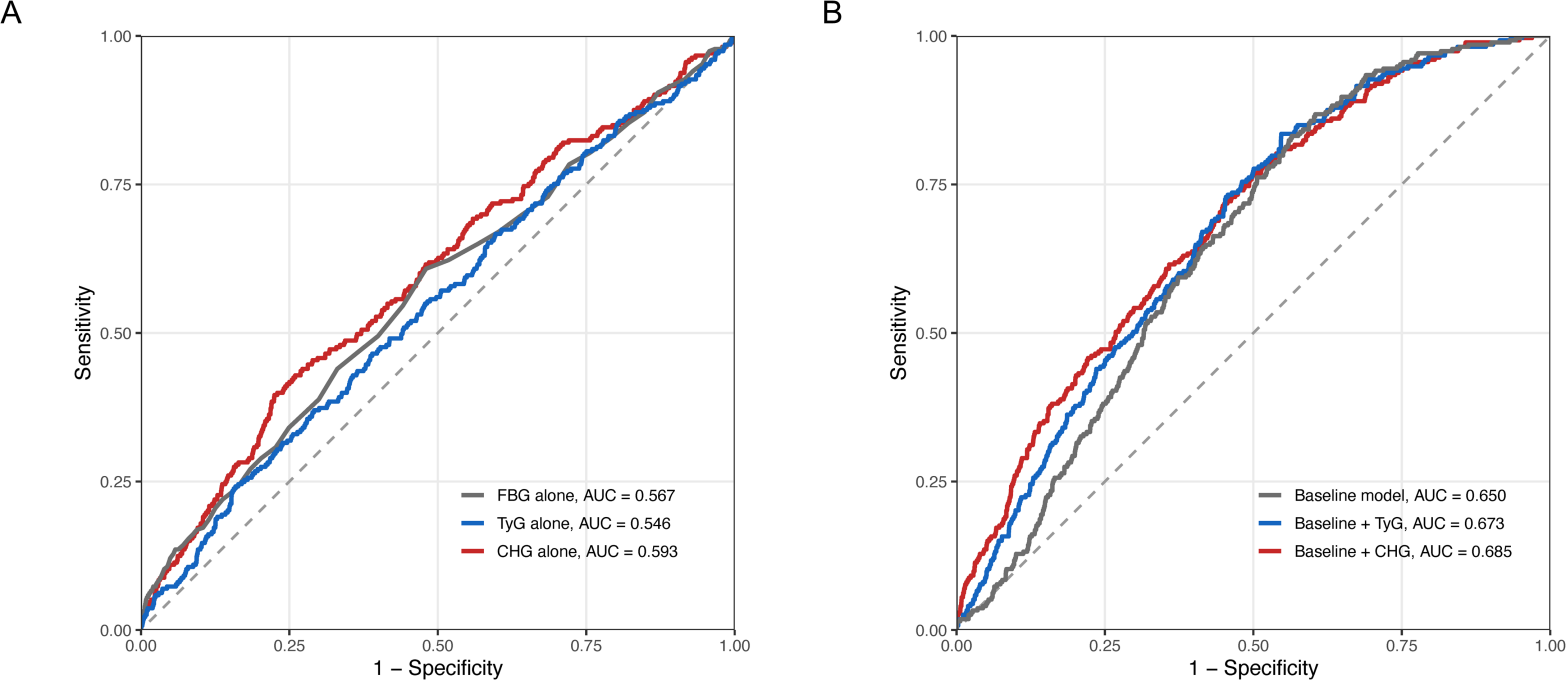
Discriminative performance of TyG and CHG in the NHANES discovery cohort. **(A)** Receiver operating characteristic (ROC) curves for FBG, TyG, and CHG as single markers. **(B)** ROC curves for the baseline model and the baseline model additionally including TyG or CHG. The baseline model included age, body mass index (BMI), systolic blood pressure (SBP), and diastolic blood pressure (DBP). FBG, fasting blood glucose; TyG, triglyceride-glucose index; CHG, cholesterol-high-density lipoprotein-glucose index; ROC, receiver operating characteristic; AUC, area under the curve.

In the NHANES discovery cohort, discriminative performance was evaluated using ROC curves (**Figure 3**). In the single-marker comparison (**Figure 3A**), CHG yielded the highest AUC among the three markers, with an AUC of 0.593 (95% CI, 0.557–0.630), compared with 0.567 (95% CI, 0.531–0.604) for FBG and 0.546 (95% CI, 0.510–0.583) for TyG.

In the incremental model comparison (**Figure 3B**), the baseline model, including age, BMI, SBP, and DBP, yielded an AUC of 0.650 (95% CI, 0.622–0.677). The addition of TyG increased the AUC to 0.673 (95% CI, 0.644–0.702), whereas the addition of CHG increased the AUC to 0.685 (95% CI, 0.655–0.716). Overall, CHG showed numerically higher discriminative performance than TyG both as a single marker and when added to the baseline model.

### 3.4 Additional supportive and sensitivity analyses in NHANES

Supportive analyses restricted to currently pregnant NHANES participants from 2007–2012 are summarized in **Supplementary Tables 1**, **2**. Among 30,442 participants in the merged 2007–2012 dataset, 182 were identified as currently pregnant, of whom 180 remained after excluding women with known diabetes or glucose-lowering medication use. After further restricting to those with available fasting glucose, TG, TC, HDL-C, age, and BMI, 77 participants were included in the final weighted main analytic sample; 56 participants had additional trimester or pregnancy-month information available.

In these currently pregnant NHANES participants, survey-weighted supportive analyses showed directionally positive associations for both TyG and CHG with proxy-defined gestational fasting dysglycemia, but the estimates were imprecise and not statistically significant across Models 1–4 (**Supplementary Table 2**).

Additional sensitivity analyses in the NHANES discovery cohort further adjusted for continuous fasting blood glucose (**Supplementary Table 3**). After this adjustment, the association for TyG was attenuated and no longer statistically significant, whereas CHG remained significantly associated in both models.

### 3.5 Baseline characteristics of the clinical validation cohort

Baseline characteristics of the clinical validation cohort are summarized in **Table 3**. Gestational age at blood sampling was similar between the non-GDM and GDM groups (11.96 ± 1.83 weeks overall; 12.08 ± 1.76 vs 11.86 ± 1.88 weeks, P = 0.378). Compared with women without GDM, those with GDM had higher BMI, SBP, and DBP (all P < 0.05). In laboratory measurements, the GDM group had higher fasting blood glucose, total cholesterol, triglycerides, TyG index, and CHG index, and lower albumin levels (all P < 0.05). No statistically significant between-group differences were observed for age, ALT, AST, creatinine, ALP, HbA1c, LDL-C, or HDL-C (all P > 0.05).

**Table 3:** Baseline characteristics of the clinical validation cohort.

Variable	Overall (n = 217)	Non-GDM (n = 101)	GDM (n = 116)	P value
Age, years	33.16 ± 4.41	33.48 ± 4.24	32.88 ± 4.55	0.319
BMI, kg/m²	29.30 ± 4.91	28.55 ± 4.48	29.96 ± 5.19	0.033
SBP, mmHg	126.04 ± 14.77	122.71 ± 13.25	128.94 ± 15.46	0.002
DBP, mmHg	80.63 ± 10.12	78.03 ± 8.69	82.90 ± 10.75	<0.001
ALT, U/L	14.80 ± 42.57	15.70 ± 59.78	14.02 ± 17.17	0.787
AST, U/L	18.95 ± 22.77	18.64 ± 30.39	19.22 ± 13.07	0.86
ALB, g/L	34.56 ± 2.55	34.98 ± 2.43	34.19 ± 2.60	0.021
Creatinine, μmol/L	46.29 ± 14.38	46.88 ± 18.27	45.79 ± 9.88	0.592
ALP, IU/L	161.74 ± 71.30	165.92 ± 87.72	158.09 ± 53.19	0.436
FBG, mg/dL	102.36 ± 24.02	97.93 ± 20.10	106.22 ± 26.46	0.009
HbA1c, %	5.51 ± 0.48	5.45 ± 0.60	5.57 ± 0.34	0.074
TC, mg/dL	216.54 ± 46.44	208.67 ± 44.35	223.39 ± 47.32	0.019
TG, mg/dL	303.83 ± 194.71	253.55 ± 109.03	347.61 ± 238.16	<0.001
LDL-C, mg/dL	129.08 ± 32.35	129.34 ± 33.05	128.84 ± 31.87	0.911
HDL-C, mg/dL	62.95 ± 17.96	64.07 ± 17.69	61.98 ± 18.21	0.394
TyG index	9.48 ± 0.62	9.30 ± 0.58	9.64 ± 0.61	<0.001
CHG index	5.17 ± 0.29	5.07 ± 0.25	5.25 ± 0.29	<0.001
Gestational age, weeks	11.96 ± 1.83	12.08 ± 1.76	11.86 ± 1.88	0.378

Data are presented as mean ± standard deviation. Gestational age refers to gestational age at blood sampling. P values were calculated using two-sample t tests.

### 3.6 Associations of TyG and CHG with GDM in the clinical validation cohort

Associations of TyG and CHG with GDM in the clinical validation cohort are presented in **Table 4**. When analyzed as continuous variables, both indices were positively associated with GDM across all models. In the fully adjusted model (Model 4), the OR was 2.45 (95% CI, 1.44–4.15) for TyG and 10.30 (95% CI, 3.14–33.79) for CHG.

**Table 4:** Associations of TyG and CHG with GDM in the clinical validation cohort.

Category	Model 1		Model 2		Model 3		Model 4	
	OR (95% CI)	P value	OR (95% CI)	P value	OR (95% CI)	P value	OR (95% CI)	P value
TyG index								
Continuous	2.73 (1.63-4.55)	<0.001	2.65 (1.58-4.45)	<0.001	2.47 (1.46-4.18)	<0.001	2.45 (1.44-4.15)	<0.001
Quartile Q1	Ref	–	Ref	–	Ref	–	Ref	–
Quartile Q2	1.63 (0.76-3.53)	0.213	1.40 (0.63-3.09)	0.407	1.46 (0.65-3.29)	0.36	1.43 (0.63-3.23)	0.387
Quartile Q3	4.12 (1.85-9.17)	<0.001	3.69 (1.64-8.30)	0.002	3.60 (1.57-8.26)	0.003	3.65 (1.58-8.41)	0.002
Quartile Q4	3.49 (1.59-7.67)	0.002	3.31 (1.49-7.33)	0.003	2.92 (1.30-6.56)	0.01	2.84 (1.26-6.40)	0.012
CHG index								
Continuous	13.52 (4.25-43.06)	<0.001	11.92 (3.65-38.92)	<0.001	10.32 (3.15-33.84)	<0.001	10.30 (3.14-33.79)	<0.001
Quartile Q1	Ref	–	Ref	–	Ref	–	Ref	–
Quartile Q2	3.33 (1.50-7.42)	0.003	3.21 (1.43-7.19)	0.005	2.74 (1.20-6.27)	0.017	2.74 (1.20-6.27)	0.017
Quartile Q3	3.33 (1.50-7.42)	0.003	3.36 (1.50-7.50)	0.003	2.89 (1.27-6.54)	0.011	2.89 (1.27-6.56)	0.011
Quartile Q4	8.41 (3.56-19.90)	<0.001	7.59 (3.17-18.18)	<0.001	6.58 (2.70-16.06)	<0.001	6.59 (2.70-16.11)	<0.001

Model 1, crude. Model 2, adjusted for age and BMI. Model 3, further adjusted for SBP and DBP. Model 4, further adjusted for gestational week. Quartile analyses used Q1 as the reference group. OR, odds ratio; CI, confidence interval.

When analyzed by quartiles, TyG showed higher odds of GDM in Q3 and Q4 relative to Q1 across Models 1–4, whereas no significant difference was observed for Q2. In Model 4, compared with Q1, the ORs were 1.43 (95% CI, 0.63–3.23) for Q2, 3.65 (95% CI, 1.58–8.41) for Q3, and 2.84 (95% CI, 1.26–6.40) for Q4.

For CHG, higher odds of GDM were observed from Q2 onward across all models. In the fully adjusted model, compared with Q1, the ORs were 2.74 (95% CI, 1.20–6.27) for Q2, 2.89 (95% CI, 1.27–6.56) for Q3, and 6.59 (95% CI, 2.70–16.11) for Q4.

### 3.7 Discrimination, calibration, and clinical utility of TyG and CHG in the clinical validation cohort

**Figure 4:**
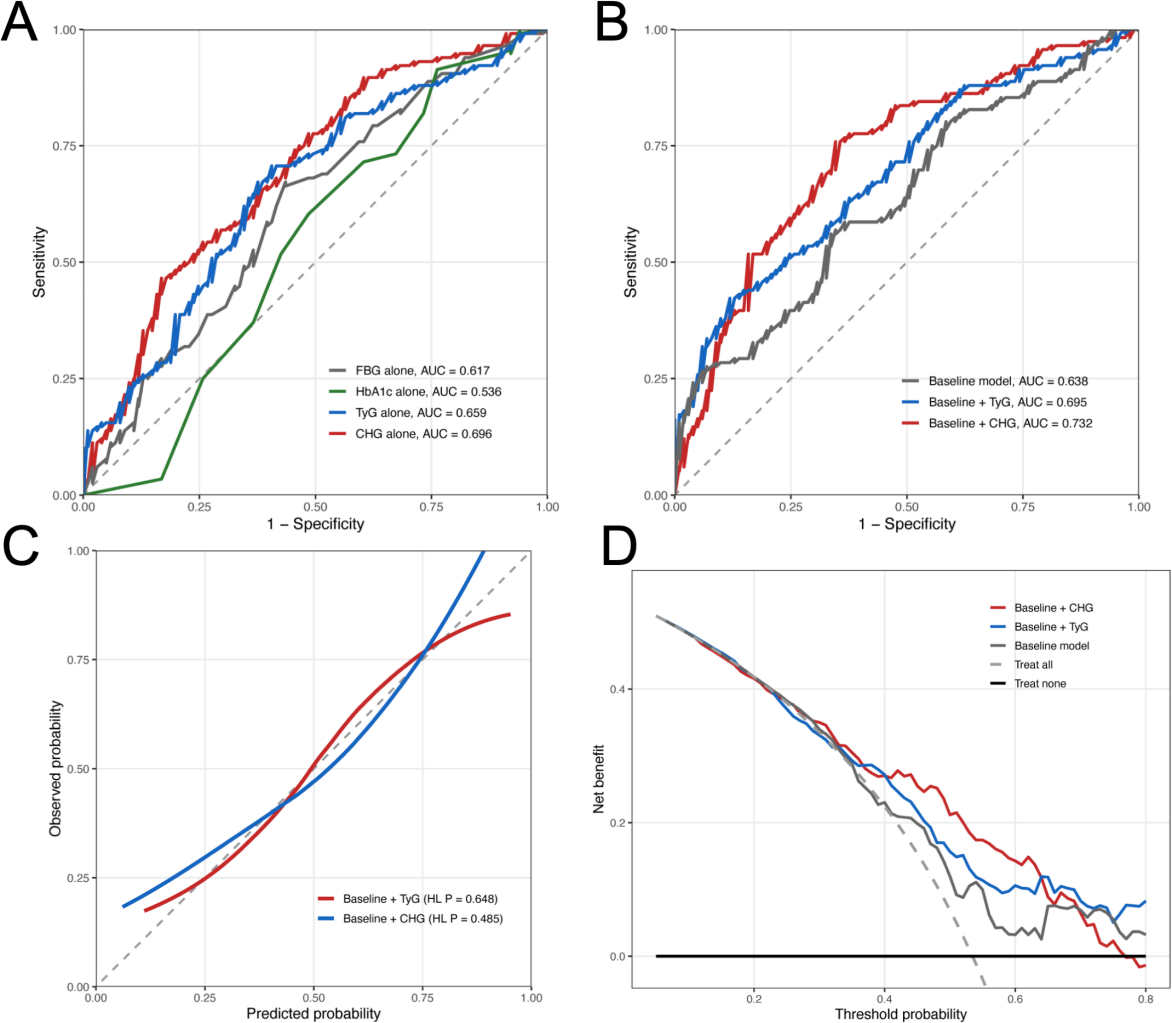
Discrimination, calibration, and clinical utility of TyG and CHG in the clinical validation cohort. **(A)** ROC curves for FBG, HbA1c, TyG, and CHG as single markers. **(B)** ROC curves for the baseline model and the baseline model plus TyG or CHG. **(C)** Calibration curves for the baseline model plus TyG and the baseline model plus CHG. **(D)** Decision curve analysis for the baseline model, the baseline model plus TyG, and the baseline model plus CHG.

Discriminative performance in the clinical validation cohort was evaluated using ROC curves (**Figures 4A, B**). In the single-marker comparison (**Figure 4A**), CHG yielded the highest AUC among the four markers, with an AUC of 0.696 (95% CI, 0.625–0.766), compared with 0.659 (95% CI, 0.586–0.732) for TyG, 0.617 (95% CI, 0.541–0.692) for FBG, and 0.536 (95% CI, 0.457–0.615) for HbA1c.

In the incremental model comparison (**Figure 4B**), the baseline model, including age, BMI, SBP, and DBP, yielded an AUC of 0.638 (95% CI, 0.564–0.711). The addition of TyG increased the AUC to 0.695 (95% CI, 0.626–0.764), whereas the addition of CHG increased the AUC to 0.732 (95% CI, 0.665–0.799).

Calibration plots are shown in **Figure 4C**. The Hosmer–Lemeshow P values were 0.648 for the baseline model plus TyG and 0.485 for the baseline model plus CHG.

Decision curve analysis is presented in **Figure 4D**. Across a range of threshold probabilities, both the baseline model plus TyG and the baseline model plus CHG showed higher net benefit than the baseline model alone. Over much of the evaluated threshold range, the baseline model plus CHG curve lay above the baseline model plus TyG curve.

Restricted cubic spline analyses for the clinical validation cohort are shown in **Supplementary Figure 1**. For both TyG and CHG, the overall associations with GDM were statistically significant, whereas no significant nonlinearity was observed. Clinical impact curves are shown in **Supplementary Figure 2**, which present the numbers of individuals classified as high risk and the corresponding true positive cases across threshold probabilities for the baseline model plus TyG and the baseline model plus CHG.

## 4 Discussion

In this two-cohort study, we performed a head-to-head comparison of the CHG and TyG indices across a survey-weighted NHANES discovery cohort and an independent clinical validation cohort. Overall, both indices were associated with dysglycemia-related outcomes across the two cohorts, whereas CHG generally showed larger effect estimates and numerically better discrimination than TyG in both cohorts. However, the absolute discrimination remained modest, and our findings are better interpreted as supporting CHG as a potential risk marker rather than a standalone screening or diagnostic tool.

The TyG index is a widely used surrogate marker of insulin resistance and has been repeatedly linked to dysglycemia and GDM risk in prior studies (10–13, 17, 18). In our study, TyG was associated with self-reported GDM history in the NHANES discovery cohort and with clinically diagnosed GDM in the validation cohort. Restricted cubic spline analyses showed significant overall associations in both cohorts, without significant evidence of nonlinearity. In the clinical validation cohort, TyG also remained associated with GDM after additional adjustment for gestational week, supporting the stability of this relationship within the available data structure. At the same time, when continuous fasting blood glucose was added to the NHANES models, the TyG association was attenuated and no longer statistically significant. This finding suggests that, in the discovery cohort, a substantial part of the TyG signal may be driven by its glycemic component.

Compared with TyG, CHG generally showed stronger associations in the primary models of both cohorts. In the NHANES discovery cohort, CHG remained significantly associated after additional adjustment for continuous fasting blood glucose, whereas TyG did not. In the clinical validation cohort, CHG also retained its association after gestational-week adjustment, both as a continuous variable and across quartile-based analyses. These findings support the possibility that incorporating cholesterol-related information may provide additional metabolic information beyond fasting glucose and triglycerides alone. A biologically plausible interpretation is that total cholesterol and HDL-C reflect complementary aspects of lipid handling and metabolic status that are not captured by triglycerides alone, particularly in pregnancy, a physiological state characterized by substantial remodeling of lipid metabolism (19–25). Although the present study was not designed to establish mechanisms, the persistence of the CHG association after additional FBG adjustment in NHANES is consistent with the view that CHG may capture information beyond fasting glycemia alone.

From a discrimination perspective, CHG showed numerically higher AUCs than TyG in both cohorts, both as a single marker and when added to a clinical baseline model. In the NHANES discovery cohort, the AUCs were 0.593 for CHG and 0.546 for TyG as single markers, and 0.685 versus 0.673 when added to the baseline model. In the clinical validation cohort, the corresponding AUCs were 0.696 versus 0.659 as single markers and 0.732 versus 0.695 when added to the baseline model. These differences were directionally consistent, but the overall discrimination remained in the poor-to-fair range rather than at a level that would support independent clinical screening use. Accordingly, our results should not be interpreted as showing that CHG is sufficient as a single predictive test. Instead, the more appropriate interpretation is that CHG may offer incremental value for early risk assessment when used alongside conventional clinical information.

The analyses of calibration and decision utility in the clinical validation cohort provide additional context for the potential clinical use of these indices. Both baseline-plus-TyG and baseline-plus-CHG models showed acceptable calibration, and both yielded higher net benefit than the baseline model alone across a range of threshold probabilities. Over much of the evaluated threshold range, the baseline-plus-CHG curve lay above the baseline-plus-TyG curve in the decision curve analysis. These findings suggest that CHG may be useful for identifying women who could merit closer monitoring or earlier preventive attention. However, because the validation cohort was derived from a tertiary metabolic disease center with a GDM prevalence of 53.5%, the apparent net benefit and clinical impact shown by DCA and CIC should be interpreted cautiously. In lower-risk primary obstetric settings, where disease prevalence is substantially lower, the balance between true positives and false positives may differ, and the transportability of these decision-analytic findings requires further external validation.

Several methodological considerations are important when interpreting the NHANES findings. First, the primary NHANES discovery outcome was self-reported GDM history rather than OGTT-confirmed GDM. Therefore, the discovery analysis should be interpreted as an evaluation of associations with a history-based dysglycemia-related outcome rather than as direct prediction of clinically confirmed GDM. Because this outcome was based on self-report, the NHANES findings should also be interpreted cautiously in light of possible recall error, outcome misclassification, and reduced clinical specificity. To partially address this limitation, we conducted an additional supportive analysis restricted to currently pregnant NHANES participants from 2007–2012, in whom proxy-defined gestational fasting dysglycemia was defined using FBG ≥92 mg/dL. Although these supportive analyses showed directionally positive associations for both TyG and CHG, the estimates were imprecise and not statistically significant, largely because of the very limited sample size. Thus, these currently pregnant NHANES analyses should be viewed as supportive rather than confirmatory.

Second, because fasting glucose is a component of both TyG and CHG, outcome-exposure circularity was a legitimate concern in NHANES-based analyses. We therefore conducted additional sensitivity analyses with explicit adjustment for continuous fasting blood glucose. In those models, CHG remained significantly associated, whereas TyG did not, which materially strengthened the interpretation that CHG may contain additional lipid-related information not fully reducible to fasting glucose alone. Third, gestational timing is an important source of potential confounding in pregnancy studies. Exact gestational week at biomarker sampling was available in the clinical validation cohort and was incorporated into sensitivity analyses, in which the associations for both indices, particularly CHG, remained directionally stable. By contrast, exact gestational week was not standardized or broadly available in the primary NHANES discovery cohort, which remains an important limitation despite our supportive analyses in currently pregnant participants.

Additional limitations should be acknowledged. The NHANES component was observational and cross-sectional in structure with respect to biomarker assessment, and the clinical validation cohort was retrospective; therefore, causal inference cannot be made. Medication exposure and baseline metabolic comorbidity may also have influenced the observed associations, although women with known diabetes and glucose-lowering medication use were excluded where possible. Residual confounding and misclassification cannot be fully excluded, particularly in NHANES, where the outcome was history based rather than OGTT confirmed. In addition, the clinical validation cohort was recruited from a specialized center and therefore represents a higher-risk population than routine community obstetric care. These factors may limit direct generalizability, especially for discrimination and decision-analytic performance estimates.

Despite these limitations, the study has several strengths. We used a dual-cohort design, applied survey-weighted analyses in NHANES, incorporated gestational-week-adjusted sensitivity analyses in the validation cohort, and directly examined the extent to which the NHANES findings were dependent on fasting glucose through additional FBG-adjusted models. The overall pattern of results was directionally consistent across cohorts, and CHG generally showed stronger effect estimates and numerically higher discrimination than TyG.

In conclusion, both TyG and CHG were associated with gestational dysglycemia/GDM-related outcomes in this study. Across the discovery and validation cohorts, CHG generally showed stronger associations and numerically better discrimination than TyG, while overall model performance remained modest. These findings support CHG as a simple and potentially useful marker for early risk assessment rather than a standalone screening tool, and further prospective studies with standardized gestational timing and broader external validation are needed.

## Data Availability

The raw data supporting the conclusions of this article will be made available by the authors, without undue reservation.

## Supplementary material

Supplementary Figure 1 Dose–response associations of TyG and CHG with GDM in the clinical validation cohort. **(A)** Restricted cubic spline curve for TyG. **(B)** Restricted cubic spline curve for CHG.

Supplementary Figure 2 Clinical impact curves in the clinical validation cohort. Clinical impact curves for the baseline model plus TyG and the baseline model plus CHG.
