# Supplementary figures and images for: Comparative predictive value of the cholesterol-high-density lipoprotein-glucose index versus the triglyceride-glucose index for gestational dysglycemia: a two-cohort study
Source: Front Endocrinol (Lausanne). 2026 Apr 27;17:1801546. doi: 10.3389/fendo.2026.1801546 (PMC13158057; doi:10.3389/fendo.2026.1801546)

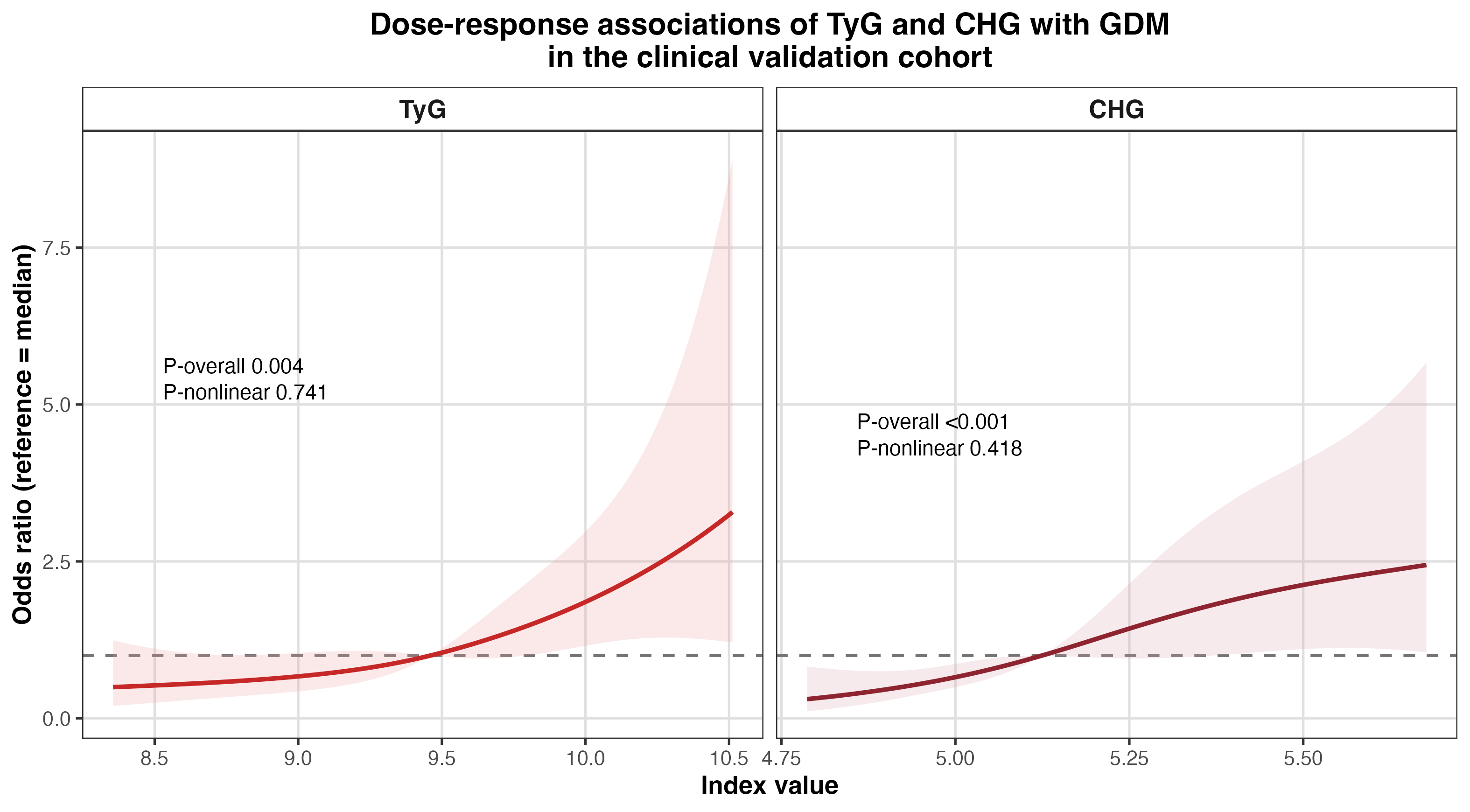

Supplement: Supplementary Figure 1 — Dose–response associations of TyG and CHG with GDM in the clinical validation cohort. (A) Restricted cubic spline curve for TyG. (B) Restricted cubic spline curve for CHG. [file Image1.png]

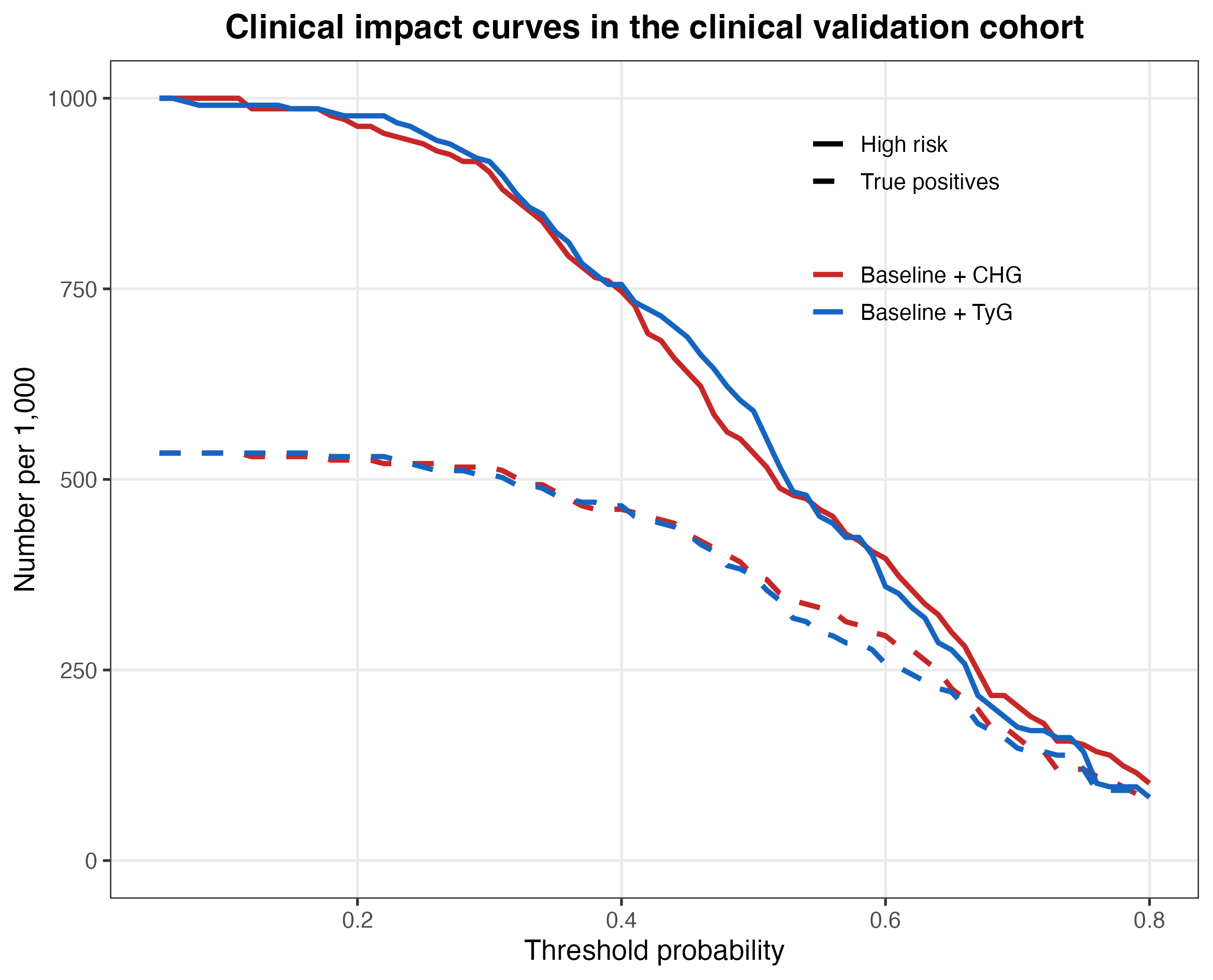

Supplement: Supplementary Figure 2 — Clinical impact curves in the clinical validation cohort. Clinical impact curves for the baseline model plus TyG and the baseline model plus CHG. [file Image2.png]
