# Supplementary material for: Comparative predictive value of the cholesterol-high-density lipoprotein-glucose index versus the triglyceride-glucose index for gestational dysglycemia: a two-cohort study
Source: Front Endocrinol (Lausanne). 2026 Apr 27;17:1801546. doi: 10.3389/fendo.2026.1801546 (PMC13158057; doi:10.3389/fendo.2026.1801546)
Supplement: Supplementary file 3 [file Table1.docx]

TableS1. Flow of currently pregnant NHANES participants included in the supportive analyses.

| step | n |
| --- | --- |
| All merged participants (2007-2012) | 30442 |
| Current pregnant women | 182 |
| Exclude known diabetes / diabetes meds | 180 |
| Has FBG + TG + TC + HDL | 78 |
| Has age + BMI | 77 |
| Has trimester information | 56 |
| Has pregnancy month information | 56 |
| Final weighted main analytic sample | 77 |
| Final weighted trimester-adjusted sample | 56 |
| Final weighted pregnancy-month sample | 56 |
