# Supplementary material for: Comparative predictive value of the cholesterol-high-density lipoprotein-glucose index versus the triglyceride-glucose index for gestational dysglycemia: a two-cohort study
Source: Front Endocrinol (Lausanne). 2026 Apr 27;17:1801546. doi: 10.3389/fendo.2026.1801546 (PMC13158057; doi:10.3389/fendo.2026.1801546)
Supplement: Supplementary file 4 [file Table2.docx]

**Table S2.** Associations of the TyG and CHG indices with proxy-defined gestational fasting dysglycemia in currently pregnant NHANES participants (2007–2012).

|  | **Model 1** | | **Model 2** | | **Model 3** | | **Model 4** | |
| --- | --- | --- | --- | --- | --- | --- | --- | --- |
| Variable | OR (95% CI) | P value | OR (95% CI) | P value | OR (95% CI) | P value | OR (95% CI) | P value |
| TyG index | 1.06 (0.42–2.66) | 0.901 | 0.98 (0.34–2.84) | 0.964 | 2.39 (0.18–32.13) | 0.527 | 5.19 (0.39–69.02) | 0.244 |
| CHG index | 7.63 (1.05–55.58) | 0.060 | 7.70 (0.78–76.49) | 0.100 | 2.29 (0.17–31.18) | 0.548 | 2.69 (0.20–36.39) | 0.476 |

Proxy-defined gestational fasting dysglycemia was defined as fasting blood glucose ≥92 mg/dL. Model 1 was unadjusted. Model 2 was adjusted for age and BMI. Model 3 was adjusted for age, BMI, and trimester. Model 4 was adjusted for age, BMI, and pregnancy month. Survey-weighted logistic regression was used. Because of the limited sample size, these analyses were interpreted as supportive rather than primary. TyG, triglyceride-glucose; CHG, cholesterol-high-density lipoprotein-glucose; OR, odds ratio; CI, confidence interval; BMI, body mass index.
