# Supplementary material for: Comparative predictive value of the cholesterol-high-density lipoprotein-glucose index versus the triglyceride-glucose index for gestational dysglycemia: a two-cohort study
Source: Front Endocrinol (Lausanne). 2026 Apr 27;17:1801546. doi: 10.3389/fendo.2026.1801546 (PMC13158057; doi:10.3389/fendo.2026.1801546)
Supplement: Supplementary file 5 [file Table3.docx]

| **Variable** | **Model A OR (95% CI)** | **P value** | **Model B OR (95% CI)** | **P value** |
| --- | --- | --- | --- | --- |
| TyG index | 1.33 (0.99–1.78) | 0.058 | 1.32 (0.99–1.77) | 0.061 |
| CHG index | 2.64 (1.55–4.48) | <0.001 | 2.60 (1.54–4.40) | <0.001 |

Table S3. Sensitivity analyses additionally adjusting for continuous fasting blood glucose in the NHANES discovery cohort

Model A was adjusted for age, BMI, and continuous fasting blood glucose. Model B was additionally adjusted for SBP and DBP. OR, odds ratio; CI, confidence interval.
